# Identification and Characterization of a Novel Porin Family Highlights a Major Difference in the Outer Membrane of Chlamydial Symbionts and Pathogens

**DOI:** 10.1371/journal.pone.0055010

**Published:** 2013-01-31

**Authors:** Karin Aistleitner, Christian Heinz, Alexandra Hörmann, Eva Heinz, Jacqueline Montanaro, Frederik Schulz, Elke Maier, Peter Pichler, Roland Benz, Matthias Horn

**Affiliations:** 1 Department of Microbial Ecology, University of Vienna, Vienna, Austria; 2 Rudolf-Virchow-Center, Deutsche Forschungsgemeinschaft - Research Center for Experimental Biomedicine, University of Würzburg, Würzburg, Germany; 3 Christian Doppler Laboratory for Mass Spectrometry, Vienna, Austria; 4 School of Engineering and Science, Jacobs University Bremen, Bremen, Germany; University of Lausanne, Switzerland

## Abstract

The *Chlamydiae* constitute an evolutionary well separated group of intracellular bacteria comprising important pathogens of humans as well as symbionts of protozoa. The amoeba symbiont *Protochlamydia amoebophila* lacks a homologue of the most abundant outer membrane protein of the *Chlamydiaceae*, the major outer membrane protein MOMP, highlighting a major difference between environmental chlamydiae and their pathogenic counterparts. We recently identified a novel family of putative porins encoded in the genome of *P. amoebophila* by *in silico* analysis. Two of these *Protochlamydia* outer membrane proteins, PomS (pc1489) and PomT (pc1077), are highly abundant in outer membrane preparations of this organism. Here we show that all four members of this putative porin family are toxic when expressed in the heterologous host *Escherichia coli*. Immunofluorescence analysis using antibodies against heterologously expressed PomT and PomS purified directly from elementary bodies, respectively, demonstrated the location of both proteins in the outer membrane of *P. amoebophila*. The location of the most abundant protein PomS was further confirmed by immuno-transmission electron microscopy. We could show that *pomS* is transcribed, and the corresponding protein is present in the outer membrane throughout the complete developmental cycle, suggesting an essential role for *P. amoebophila*. Lipid bilayer measurements demonstrated that PomS functions as a porin with anion-selectivity and a pore size similar to the *Chlamydiaceae* MOMP. Taken together, our results suggest that PomS, possibly in concert with PomT and other members of this porin family, is the functional equivalent of MOMP in *P. amoebophila*. This work contributes to our understanding of the adaptations of symbiotic and pathogenic chlamydiae to their different eukaryotic hosts.

## Introduction

Chlamydiae are a group of obligate intracellular bacteria with an extraordinarily broad host spectrum. They include important human pathogens like *Chlamydia* (aka *Chlamydophila*) *pneumoniae* and *Chlamydia trachomatis* as well as many animal pathogens and symbionts of amoebae [1]–[4]. All chlamydiae share a biphasic developmental cycle in which they alternate between two developmental forms, the extremely stable and infectious elementary body (EB) and the replicative reticulate body (RB) [5]. At the beginning of the developmental cycle, EBs attach to and are taken up by the host cell. Upon entry, chlamydiae reside within a host-derived vacuole [6] where the EBs differentiate into RBs. After several rounds of replication, RBs re-differentiate into EBs and leave the host cell by either lysis of the host or exocytosis in order to infect new host cells [2], [7].

During all stages of the chlamydial developmental cycle, proteins in the bacterial outer membrane play an important role. They mediate the first contact to the host cell, and once inside the host, they are involved in the uptake of nutrients and the removal of waste products. Being surface-exposed, outer membrane proteins represent promising candidates for vaccine development [8], [9] and have therefore been thoroughly studied for the pathogenic chlamydiae, which have been grouped into the family *Chlamydiaceae* [10]–[16]. The major outer membrane protein (MOMP) and the two cysteine-rich proteins OmcA and OmcB are the most abundant proteins in the outer membrane of the *Chlamydiaceae* and together form the chlamydial outer membrane complex (COMC) [17], [18]. Chlamydiae lack detectable amounts of peptidoglycan [19]. Instead, the COMC and OmcA and OmcB in particular stabilize the outer membrane by forming extensive disulfide-bonds in the osmotically stable EBs whereas these bonds are reduced in the more fragile RBs [13], [20]–[22].

MOMP is the most abundant protein in the outer membrane of the *Chlamydiaceae* and makes up about 60% of the proteins of the COMC in EBs [18]. MOMP determines *C. trachomatis* serovars [23] and functions as a diffusion porin, a group of proteins that form channels in the outer membrane of Gram-negative bacteria, facilitating passive diffusion of small molecules [24]–[26]. The porin function of MOMP was first suggested by Bavoil and coworkers based on liposome swelling assays [27] and later confirmed by lipid bilayer measurements using purified native and recombinant MOMP [28], [29]. MOMP shows a beta-barrel structure with a pore size of 2 nm [30] and occurs as trimer in the outer membrane [31].

In contrast to the *Chlamydiaceae*, little is known about the composition of the outer membrane of other chlamydiae. Several key mechanisms for host cell interaction, such as a type three secretion system and its effector proteins, are conserved among all chlamydiae [32] [33], [34]. Yet, the genome of the amoeba symbiont *Protochlamydia amoebophila* encodes no homologue of MOMP [33], and antibodies targeting *Chlamydiaceae* MOMP did not bind to the outer membrane of these bacteria [35]. A recent study identified 38 outer membrane proteins of *P. amoebophila* by combining 1D and 2D gel electrophoresis of outer membrane fractions with mass spectrometry analysis [36]. The identified proteins included OmcA (pc0617) and OmcB (pc0616). Additionally, a novel protein family was identified consisting of four proteins that share an amino acid sequence identity of 22–28% and have no functionally characterized homologues in other organisms (Table 1). Two of these proteins were frequently detected in outer membrane fractions. Both were predicted to form beta-barrels by *in silico* analysis and to contain signal peptides. Their predicted structure and their high abundance in outer membrane fractions led to the hypothesis that they function as porins and together form the COMC of *P. amoebophila* by interactions with OmcA and OmcB. Because of the lack of significant sequence similarities with other characterized proteins we propose the names PomS, PomT, PomU, and PomV (Pom for “*Protochlamydia* outer membrane protein”) for their designation.

**Table 1:** Table **1.** Members of the putative porin family of *P. amoebophila* are predicted to be localized to the outer membrane by *in silico* analysis.

	PomS	PomT	PomU	PomV
**Locus tag**	pc1489	pc1077	pc0870	pc1860
**Molecular mass in kDa**	36.3	39.0	34.4	37.5
**Signal peptide**	yes	yes	yes	yes
**Prediction of alpha-helix formation**	no	no	no	no
**Localization to outer membrane**	yes	yes	unknown	yes
**Prediction of beta-barrel formation**	yes	yes	no	yes
**Predicted lipoprotein**	no	no	no	yes
**Probability to be an OMP**	97.72%	100%	97.68%	97.22%
**pCOMP prediction**	integral outer membraneprotein (cluster 081)	integral outer membraneprotein (cluster 081)	ambiguous prediction	lipoprotein (cluster 081)
**Best blastp hit except for members** **of porin family**	hypothetical protein of *Methylophaga* sp. JAM7e-value 2e−0719.9%identity	hypothetical protein of*Methylomicrobium alcaliphilum*e-value 3e−19 17.1%identity	hypothetical protein of *Methylophaga* sp. JAM7e-value 8e−04 19.1% identity	EGF-like domain-containing protein of *Dictyostelium discoideum* e-value 0.15 8.9% identity
**Presence in outer membrane** **protein fractions ** [**36**]	yes	yes	no	yes
**Experimental evidence for outer** **membrane location in this study**	yes	yes	no	no

Signal peptides were predicted using SignalP4 [80], alpha-helix formation was predicted with TMHMM, localization to the outer membrane with Cpsortdb [81], beta-barrel formation with MCMBB, BOMP and Pred-TMBB [82], [83], lipoprotein signal peptides with LipoP [84], the probability of the localization to the outer membrane using HHOMP [85] and chlamydial outer membrane proteins with pCOMP [48].

In this study, we provide evidence that all members of the novel protein family found in *P. amoebophila* represent pore-forming proteins, and for two of them we confirmed their outer membrane location. The most abundant outer membrane protein of this family is expressed throughout the complete developmental cycle of *P. amoebophila*, and lipid bilayer measurements further confirmed its function as porin. Our study provides a first detailed analysis of outer membrane proteins of an environmental counterpart of pathogenic chlamydiae and shows that a novel porin family represents the functional equivalent of the *Chlamydiaceae* MOMP in *P. amoebophila*.

## Materials and Methods

### Cultivation of Organisms and Infection Experiments

Uninfected *Acanthamoeba castellanii* Neff or *A. castellanii* Neff infected with *P. amoebophila* were grown axenically in 10 or 150 ml TSY medium (30 g/L trypticase soy broth, 10 g/L yeast extract, pH 7.3) at 20°C. Cultures were supplied with fresh medium every one to two weeks depending on amoebal growth. *Escherichia coli* strains were grown in standard LB medium at 37°C.

For infection experiments cultures of *A. castellanii* Neff were harvested and the number of amoebae was counted using a Neubauer counting chamber. Amoebae were seeded in the wells of a multiwell dish and were infected with purified *P. amoebophila* EBs at an MOI of 5 or 10. Multiwell dishes were centrifuged at 600×g for 15 min at 20°C, and the end of centrifugation was regarded as time point 0 hours post infection (p.i.). After centrifugation, the medium was exchanged and infected cultures were grown at 20°C. At selected time points cells were fixed for immunofluorescence analysis.

### Purification of Elementary Bodies of *P. amoebophila* and Isolation of Pc1489

*P. amoebophila* EBs for infection experiments were purified as previously described [36]. Highly enriched fractions of EBs were obtained using two additional centrifugation steps to further remove host cell debris [32]. Highly purified EBs were thawed and centrifuged at 10,621×g for 15 min at 4°C. The resulting pellet was resuspended in 100 µl POP05-buffer (0.087 g/L EDTA, 5.84 g/L NaCl, 300 mM Na_x_PO_4_, 0.5% n-octly-polyoxyethylen; pH 6.5) [37] with 100 mM freshly added dithiothreitol (DTT) per 3 mg EBs (wet weight) and incubated for 1 h at 37°C on a rocking platform. Cell debris was removed by centrifugation (10,621×g, 10 min, 4°C), an equal volume of ice-cold acetone was added to the supernatant and proteins were precipitated for 1 h at −20°C. The suspension was centrifuged as before and the resulting pellet was resuspended in 400 µl Buffer A (2.9 g/L HEPES, pH 7.5, 0.292 g/L NaCl, 0.5% n-octly-polyoxyethylen). Undissolved matter was removed by centrifugation at 10,621×g for 10 min at 20°C. After equilibration of a Vivapure Q-Mini-spin column (Sartorius) with 400 µl Buffer A, the supernatant was applied onto the column and centrifuged at 2,000×g for 5 min at 20°C. The column was washed twice with Buffer A. Elution of proteins was achieved by applying a gradient with increasing NaCl concentrations ranging from 0.1 to 1 M NaCl. The flow-through of all steps was collected. 4×Laemmli buffer without DTT was added to the samples and samples were run on a 12.5% SDS-PAGE gel, followed by staining with colloidal Coomassie (100 g/L (NH_4_)_2_SO_4,_ 20 g/L orthophosphoric acid, 25% methanol, 0.625 g/L Coomassie Brilliant Blue G-250). Fractions that showed only a single band for Pc1489 at the correct size were collected and pooled. To concentrate samples, proteins were precipitated with ice-cold acetone and resuspended in Buffer A without n-octyl-polyoxyethylen (n-octyl-POE). Protein concentrations were determined using the BCA™ Protein Assay Kit (Pierce Biotechnology). The identity of the purified protein was confirmed by SDS-PAGE analysis in combination with mass spectrometry.

### Mass Spectrometry Analysis

Sample processing and high-performance liquid chromatography mass spectrometry (HPLC-MS) analysis were perfomed as described previously [36]. Briefly, bands were excised from the gel, reduced with DTT, alkylated with iodoacetamide and digested overnight with trypsin. Samples were separated on an Ultimate plus HPLC system (Dionex) coupled online to an LTQ mass spectrometer (Thermo Scientific). Raw files were searched with ProteomeDiscoverer 1.3.0.339 and Mascot 2.2 using the following settings: trypsin/P, maximum 2 missed cleavage sites, 1.5 Da precursor ion tolerance, 0.8 Da fragment ion tolerance, cabamidomethyl-cysteine as fixed modification, oxidation of methionine and deamidation of asparagine or glutamine as variable modifications. The fasta database comprised sequences from *P. amoebophila* as well as proteins of the amoeba host and contaminants. Proteins were quantified by calculating spectral counts and normalized spectral abundance factor (NSAF) for all *P. amoebophila* proteins identified with p<0.05 [38]. As the precision of spectral counting was shown to be optimal for proteins with five or more spectra, calculation of NSAF was restricted to proteins with a minimum of five spectra with a Mascot score of 20 or above [39]. NSAF values were converted to percentages as a measure of relative abundance.

### Transcriptional Analysis

For the isolation of RNA, 3 ml of *A. castellanii* infected with *P. amoebophila* were harvested by centrifugation (7,323×g, 10 min). The pellet was resuspended in 750 µl Trizol and cells were disrupted using a bead beater at an intensity of 4.5 for 30 sec. The cell suspension was then centrifuged at 12,000×g for 5 min. The supernatant was incubated at room temperature for 5 min followed by the addition of 200 µl chloroform. The solution was shaken vigorously for 15 sec, further incubated for 5 min and finally centrifuged at 12,000×g for 15 min at 4°C. The supernatant was taken off carefully, mixed with 500 µl isopropyl alcohol per 750 µl volume and incubated at room temperature for 10 min. After another centrifugation step (12,000×g, 10 min, 4°C), the resulting RNA pellet was washed with 1 ml 75% ethanol and centrifuged for 5 min at 7,000×g and 4°C. The pellet was air-dried for 10 min and dissolved in 30 µl distilled water treated with diethylpyrocarbonate (DEPC). Isolated RNA was quantified using a NanoDrop® ND-1000 spectral photometer and either stored at −80°C or treated directly with DNaseI (Sigma-Aldrich). DNA was digested by adding 1 U DNaseI/2 µg RNA and incubation at room temperature for 1 h. RNA was precipitated with pure ethanol at −20°C overnight and resolved in DEPC-treated distilled water. RNA quality was assessed by agarose gel electrophoresis. To test for DNA still present in the RNA preparations, PCR without reverse transcription was performed and no product was obtained (data not shown). Three independent biological replicates of RNA isolated at 0 h p.i., 24 h p.i., 48 h p.i. and 96 h p.i were analyzed.

Reverse transcription was performed using the RevertAid™ First Strand cDNA Kit (Fermentas). 1 µg RNA was applied per reaction and random hexamer primers were used for reverse transcription. A negative control without reverse transcriptase was performed in parallel. The RT conditions were 5 min at 25°C, 60 min at 37°C and 5 min at 70°C. The obtained cDNA was stored at −20°C until further use. Primers targeting *pc1489* and the 16S rRNA gene of *P. amoebophila* were designed using the online tool Primer3plus [40] (Table S1). Primer concentrations and annealing temperatures were optimized using gradient PCR. Genes were cloned into the pCR-XL-TOPO vector (Invitrogen) and these constructs were used as standards for calibration of the PCR assay. The Platinum® SYBR® Green qPCR SuperMix-UDG Kit (Invitrogen) was used for amplification following the manufacturer’s instructions by applying 1 µl cDNA to the reaction mixture. All standards and samples were analyzed on one plate during a single run using an iCycler (Bio-Rad). Each plate included three different negative controls: a no template control (where all the reaction reagents except for cDNA were used), an RNA only control (to test for residual chlamydial DNA) and cDNA obtained from uninfected amoebae. The qPCR program was: denaturation at 95°C for 3 min, followed by 35 cycles of 40 sec at 95°C, 30 sec at 60°C and 30 sec at 72°C. Final elongation was allowed for 1 min at 72°C, followed by 30 sec at 95°C and a melting curve from 55–95°C. For interpretation and further analysis of the qPCR results the iCycler software was used. Copy numbers of *pc1489* were adjusted to the number of organisms present at the different stages of the developmental cycle by normalization with copy numbers of the 16S rRNA gene at the respective time point [41]. Analysis of the melting curves of the PCR products for *pc1489* and the 16S rRNA showed a single-peak for the amplicons, indicating that a single PCR product was formed. This was confirmed by analysis of the products on a 2% agarose gel. The negative controls did not show any amplification product (data not shown).

### Cloning and Heterologous Expression of Proteins in *E. coli*

Genes encoding the predicted porins were cloned as full length copies or without the predicted signal peptide using two different cloning vectors. Full length *pc0870*, *pc1077*, and *pc1860* were cloned into the BamHI and NdeI restriction sites of the vector pet16b (Novagen). Full length *pc1489* was cloned into the XhoI site of pet16b. *Pc0870*, *pc1077* and *pc1489* were cloned into the KpnI and PstI sites of pQE-30 (Qiagen) excluding the first 21 bp encoding the predicted signal peptide. DnaK (*pc1499*), which served as control in immunofluorescence analysis, was cloned into the SmaI and PstI restriction sites of pQE-30. Genes were amplified using the High Fidelity PCR Enzyme Mix (Fermentas) or the Phusion High-Fidelity DNA Polymerase (New England BioLabs). Forward and reverse primers contained sequences to introduce recognition sites for the respective restriction enzymes. For cloning into the vector pet16b, an additional C-terminal 6× His-tag was introduced via the reverse primer to compensate for possible removal of the N-terminal His-tag by cleavage of the signal peptide. Amplified fragments were first cloned into the vector pCR-XL-TOPO (Invitrogen) and subsequently cloned into the expression vectors pet16b and pQE-30. All constructs were sequenced before transformation into *E. coli* BL21 (DE3) (Invitrogen) or *E. coli* M15 (Qiagen). Heterologous expression was induced by addition of 1 mM isopropyl β-D-1-thiogalactopyranoside (IPTG) to cultures at an OD_600_ of 0.6. Heterologously expressed Pc1077 without leader sequence and Pc1499 were purified using HisTrap purification columns (GE Healthcare Biosciences) according to the instructions of the manufacturer. The identity of the purified proteins was verified by 1D gel electrophoresis combined with mass spectrometry (data not shown).

### Toxicity Assay

To analyse the toxicity of the heterologously expressed proteins, LB medium was supplemented with 0.4% glucose to ensure repression of the T7 lac promoter of the vector pet16b. pet16b containing pc0870, pc1077, pc1489 or pc1860 was transformed into E. coli BL21 (DE3), and cells were grown on LB plates overnight at 37°C. Single colonies were inoculated in LB, and protein expression was induced by addition of 1 mM IPTG at an OD_600_ of 0.6. As controls, the vector pet16b without insert and pet16b containing a gene fragment coding for the inclusion membrane protein IncA (pc0399) of P. amoebophila were used [32]. Samples were taken at 0, 10 and 60 min after induction, they were diluted in phosphate buffered saline (PBS, 130 mM NaCl, 10 mM Na_x_PO_4_; pH 7.2–7.4) and plated on LB plates. After incubation overnight, the number of colony forming units (cfu) was counted. All experiments were performed in three biological replicates.

### Generation of Polyclonal Antibodies, Immunofluorescence, and Immunoelectron Microscopy

All antibodies used in this study were produced by Eurogentec (Belgium). To generate polyclonal antibodies against Pc1489, 400 µg of Pc1489 purified from *P. amoebophila* EBs in Buffer A without n-octyl-POE were used for immunization of one chicken. To generate antibodies against Pc1077, heterologously expressed protein without leader sequence purified from *E. coli* was used for immunization of two rabbits. To generate antibodies against DnaK, one chicken and two guinea pigs were immunized with heterologously expressed DnaK. IgY from pre-immune sera and egg yolk collections was purified using a HiTrap™ IgY Purification HP column (GE Healthcare) according to the manufacturer’s instructions. Pre-immune sera were obtained for all immunizations and tested in Western Blot and immunofluorescence analyses. To remove antibodies targeting amoeba proteins, sera were adsorbed with amoeba lysate prior to experiments [36]. Pre-immune serum and antibodies targeting Pc1489 were affinity purified on a polyvinylidene fluoride (PVDF) membrane as previously described to further reduce unspecific background signals [42]. Immunofluorescence analysis of methanol and paraformaldehyde fixed cells and immunogold labelling of ultrathin cryosections was performed as described previously [32].

### Western Blot Analysis

Cells were harvested by centrifugation (18,000×g for 2 min for *E. coli* or 7,323×g for 5 min for amoebae). Pellets were resuspended in 4× Laemmli buffer with 400 mM DTT and heated to 95°C for 5 min. Nucleic acids present in the samples were removed by digestion with the nuclease Benzonase (Novagen) for 1 h. Outer membrane fractions of *P. amoebophila* were obtained either by treatment with N-laurylsarcosine (sarkosyl) as described previously [36] or by incubation in POP05 buffer for 1 h as described above. Proteins were separated by 1D gel electrophoresis, transferred to a PVDF membrane (GE Healthcare) by semi dry blotting in transfer buffer (5.8 g/L Tris, 2.9 g/L glycine, 20% methanol) using a Trans-Blot® SD Semi-Dry Electrophoretic Transfer Cell (Bio-Rad) and proteins were detected using specific antibodies [36]. Detection of heterologously expressed Pc1077 in *E. coli* lysates by Western blot analysis proved to be difficult. The protein was not detectable in Western blot analysis after transfer with standard transfer buffer and staining of SDS-PAGE gels after blotting showed that Pc1077 did not elute from the gel (data not shown). Addition of 0.05% SDS to the buffer improved transfer, but still a substantial amount of protein could be detected in the gel after blotting and blots showed high background (data not shown). The best results were obtained with a transfer buffer containing urea (12 mM Tris, 48 mMol glycine, 5 mM NaH_2_PO_4_, 0.55% SDS, 3 M Urea) which was developed for the transfer of membrane proteins [43] and this buffer was used for the detection of Pc1077 in subsequent experiments. To test for the presence of multimers of Pc1489 in the outer membrane, outer membrane protein fractions from highly enriched EBs were cross-linked as described previously using bis(sulfosuccinimidyl)suberate [30]. Cross-linked samples were analyzed by SDS-PAGE and Western blot analysis.

### Infection Inhibition Assay

*P. amoebophila* EBs were thawed at 37°C and 1 volume of PBS was added. Heat inactivation was achieved by incubation at 56°C for 30 min [44]. Anti-Pc1489 antibodies, targeting the putative porin, and anti-Pam antibodies, targeting the immunodominant components of the outer membrane of *P. amoebophila* [32], were diluted in FA Block solution (2% bovine serum albumin in PBS) to reach final dilutions of 1∶15 and 1∶150. EBs and heat-inactivated EBs were incubated with the antibody solutions for 30 min at 37°C with mild shaking. As controls, EBs and heat inactivated EBs were incubated with FA Block solution only. The pre-incubated EBs were used to infect amoebae at an MOI of 5 and samples were fixed at 0, 6, 12, 24, 48, 72 and 96 h p. i. and stored at 4°C for later analysis. Immunofluorescence analysis was performed as described above, and for the time point 48 h p.i. the ratio of infected amoebae to all amoebae was determined by counting at least 100 amoebae. All experiments were performed in three biological replicates.

### Planar Lipid Bilayer Assays

The methods used for black lipid bilayer experiments have been described previously [45]. The black lipid bilayer instrumentation consisted of a Teflon chamber with two compartments of 5 ml volume, which were separated by a thin wall and connected by a small circular hole with an area of about 0.4 mm^2^. A 1% (w/v) solution of diphytanoyl-phosphatidylcholine (PC) (Avanti Polar Lipids) in *n*-decane was used to form the membranes across the hole. The Pc1489 (PomS)-containing protein fractions were diluted 1∶100 in 1% Genapol (Roth) and added at a concentration of about 10 ng/ml to the aqueous phase after the membrane had turned black. The membrane current was measured with a pair of Ag/AgCl electrodes with salt bridges connected in series to a voltage source and a highly sensitive current amplifier (Keithley 427). The temperature was throughout kept at 20°C. The amplified signal was recorded on a strip chart recorder. Zero-current membrane potential measurements were performed in the presence of a salt gradient as described earlier [46], [47]. After the insertion of more than 100 channels into the PC membranes, the KCl concentration (300 mM KCl) was raised 2.5-fold by addition of 3 M KCl to one side of the membrane. The zero-current membrane potentials were measured with a high impedance electrometer (Keithley 617) and analyzed using the Goldman-Hodgkin-Katz equation [46], [47].

## Results

### Toxicity of PomS, PomT, PomU, and PomV for *E. coli*

To characterize the putative novel outer membrane protein family of *P. amoebophila* [36], [48], we initially tried to clone and express PomS (*pc1489*), PomT (*pc1077)*, PomU (*pc0870*), and PomV (*pc1860*) in the heterologous host *E. coli*. Our first attempts to express the full length proteins using different expression vectors in *E. coli* failed. When protein expression was induced, the optical density (OD_600_) of the cultures decreased and no overexpression of the proteins was observed by SDS-PAGE, which indicates host cell lysis due to detrimental effects of the heterologous proteins.

**Figure 1:**
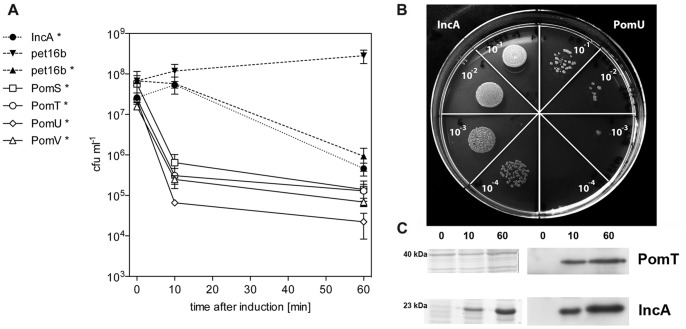
Toxic effect of the heterologous expression of PomS, PomT, PomU, and PomV in *E. coli*. (**A**) Survival of *E. coli* BL21 (DE3) carrying different pet16b plasmid constructs after induction of protein expression (labeled with *) and without induction. As controls, the vector pet16b without insert and pet16b containing a gene fragment coding for the inclusion membrane protein IncA (*pc0399*) of *P. amoebophila* were used [32]. Induction of expression of proteins of the putative porin family lead to a rapid decrease in the number of colony forming units (cfus) compared to the non-toxic protein IncA. Ten minutes after induction, the numbers of cfus of *E. coli* expressing the putative porins were significantly lower than those of all controls (p<0.05, one-way ANOVA test and Dunnett’s post test); this difference was not significant anymore at sixty minutes after induction. The mean number of cfus for three independent replicates is shown +/− the standard error of the mean (SEM). (**B**) Visualization of the toxic effect of the putative porins on *E. coli* 10 min after induction of protein expression. Colonies formed by 10 µl droplets of the same dilutions for the non-toxic IncA (left) and the putative porin PomU (right) are shown after incubation overnight at 37°C. Similar results were obtained for all four putative porins tested. Dilutions range from 1∶10 to 1∶10,000. (**C**) Detection of protein expression by SDS-PAGE (left) and Western blot analysis (right) for PomT and IncA. Time in min after induction of protein expression by addition of IPTG is indicated above the lanes. Expression of IncA can be detected by SDS-PAGE and Western blot analysis whereas expression of PomT can be detected only by the more sensitive Western blot analysis.

The overexpression of *Chlamydiaceae* MOMP in *E. coli* resulted in a strong decrease in the number of colony forming units (cfus) after induction of protein expression [49]. To investigate whether the predicted porins of *P. amoebophila* show a similar effect, we performed a time-course experiment and compared induced and uninduced *E. coli* cultures with a control protein (IncA) and an empty vector. Consistent with our initial observations, expression of the proteins PomS, PomT, PomU, and PomV had an immediate lethal effect on *E. coli* as indicated by a strong decrease in the number of cfus already 10 min after induction of protein expression. No decrease was observed at this time point when the empty vector alone or the expression of a non-toxic *P. amoebophila* protein was induced (Fig. 1A and 1B). Sixty minutes after induction, the number of cfus decreased even further for cells expressing the putative porins. At this time point also induced cells containing the control vector without an insert or encoding the non-toxic protein showed a decrease in the number of viable cells (Fig. 1A). This decrease was not as strong as for the putative porins and could result from toxicity of the expression vector for the host cells [49], [50] or from interference of the strong production of heterologous proteins with cellular processes because of the high transcription rate of the T7 RNA polymerase [51], [52].

To link the observed toxicity to protein expression, we tried to detect the heterologous proteins by SDS-PAGE. With this technique, a band for the non-toxic control protein was observed 10 and 60 min after induction, but no bands were visible for the putative porins (Fig. 1C). We therefore used the more sensitive Western blot analysis. This resulted in the detection of PomT at 10 and 60 min after induction of protein expression as well as in the detection of IncA (Fig. 1C). However, we could not detect expression of PomS, PomU, and PomV, probably due to the low amounts in which these proteins were expressed. The observed strong toxic effects on *E. coli* were thus caused by minute amounts of these proteins.

**Figure 2:**
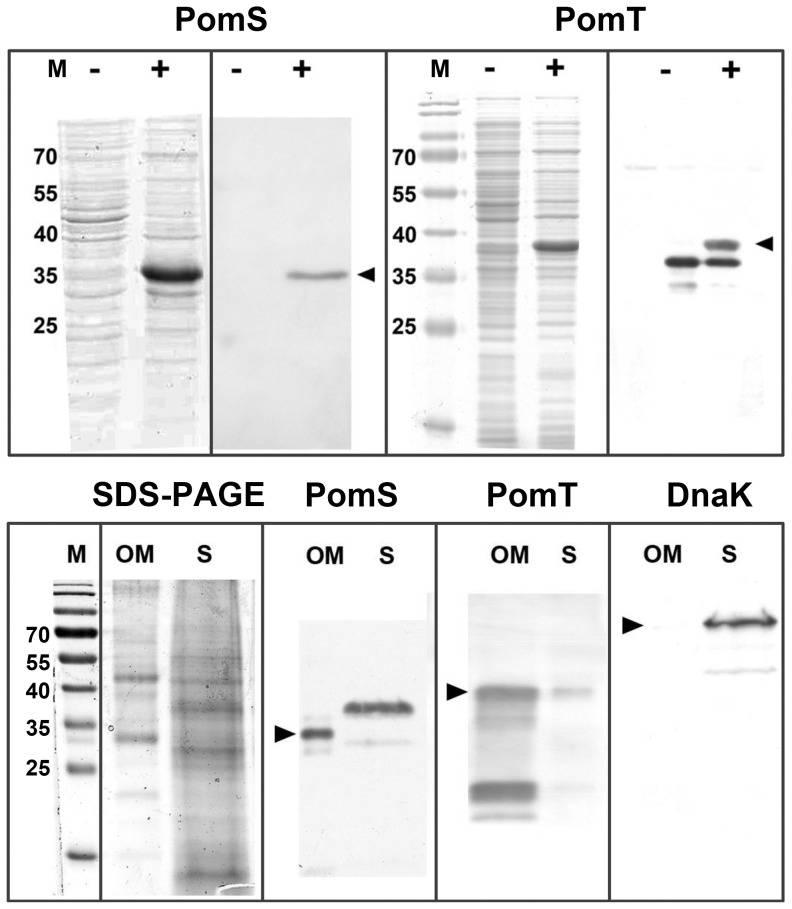
Detection of PomS and PomT after overexpresson in *E. coli* and in outer membrane fractions of *P. amoebophila* **.** Upper panel: An additional band in SDS-PAGE gels (left) is present after induction of expression of leaderless PomS or PomT in *E. coli* (lanes labeled “+”) compared to uninduced samples (lanes labeled “−”). Western blot analysis (right) using polyclonal anti-PomS and anti-PomT antibodies demonstrates specificity for the heterologously expressed proteins. The anti-PomT antibodies additionally target one *E. coli* protein with a lower molecular mass. Bands at the correct molecular mass for the leaderless proteins (33.9 kDa for PomS and 36.9 kDa for PomT) are indicated by arrow heads. Molecular mass of marker bands (M) in kDa. Lower panel: Bands for leaderless PomS (33.9 kDa) and PomT (36.9 kDa) can be observed in the sarkosyl-insoluble outer membrane fraction (OM) and in the sarkosyl-soluble (S) fraction using specific polyclonal antibodies (bands at the correct molecular mass are indicated by arrow heads). The cytoplasmic heat-shock protein DnaK, which served as a control, is detected only in the sarkosyl-soluble fraction. For comparison, a SDS gel stained with colloidal coomassie is shown on the left. Molecular mass of marker bands (M) in kDa.

Previous studies have shown that removal of the signal peptide, which prevents secretion, can help in the overexpression of porins [29]. Indeed, when we tested PomS, PomT, and PomV without the predicted leader sequence overexpression was successful for all three proteins as indicated by bands at the correct size on SDS-PAGE gels. We chose to analyse PomS and PomT in more detail, which were most abundant in *P. amoebophila* outer membrane fractions [36]. Polyclonal anti-PomS and anti-PomT antibodies recognized PomS and PomT expressed in *E. coli* resulting in a strong band at the correct molecular mass (Fig. 2, upper panel).

### Location of PomS and PomT in the Outer Membrane of *P. amoebophila*

The transport function of porins is inherently linked to their presence in the outer membrane. We therefore investigated the location of the putative porins PomS and PomT with Western blot, immunofluorescence analysis, and immuno-transmission electron microscopy (immuno-TEM). First, soluble and insoluble protein fractions of highly purified EBs after treatment with the detergent sarkosyl were analyzed by Western blot. In contrast to the cytoplasmic protein DnaK, which was absent in the sarkosyl-insoluble fraction containing proteins of the outer membrane complex, strong bands were observed for PomS and PomT (Fig. 2, lower panel). In addition, a band for PomS was also detected in the sarkosyl-soluble fraction at a higher molecular mass, possibly representing the full length protein before removal of the signal peptide. For PomT, the band detected in the sarkosyl-soluble fraction was much weaker than the band detected in the outer membrane fraction. For this protein, additional bands with lower molecular mass were observed. This is consistent with a previous study, in which PomT was detected in lower molecular bands of outer membrane protein fractions of *P. amoebophila* by mass spectrometry [36].

Most known porins function as trimers [53], including MOMP of the *Chlamydiaceae* [54]. We therefore tested for the presence of multimers of PomS by crosslinking outer membrane preparations of EBs of *P. amoebophila*, but under the conditions used we could not observe any evidence for the presence of multimers (data not shown).

**Figure 3:**
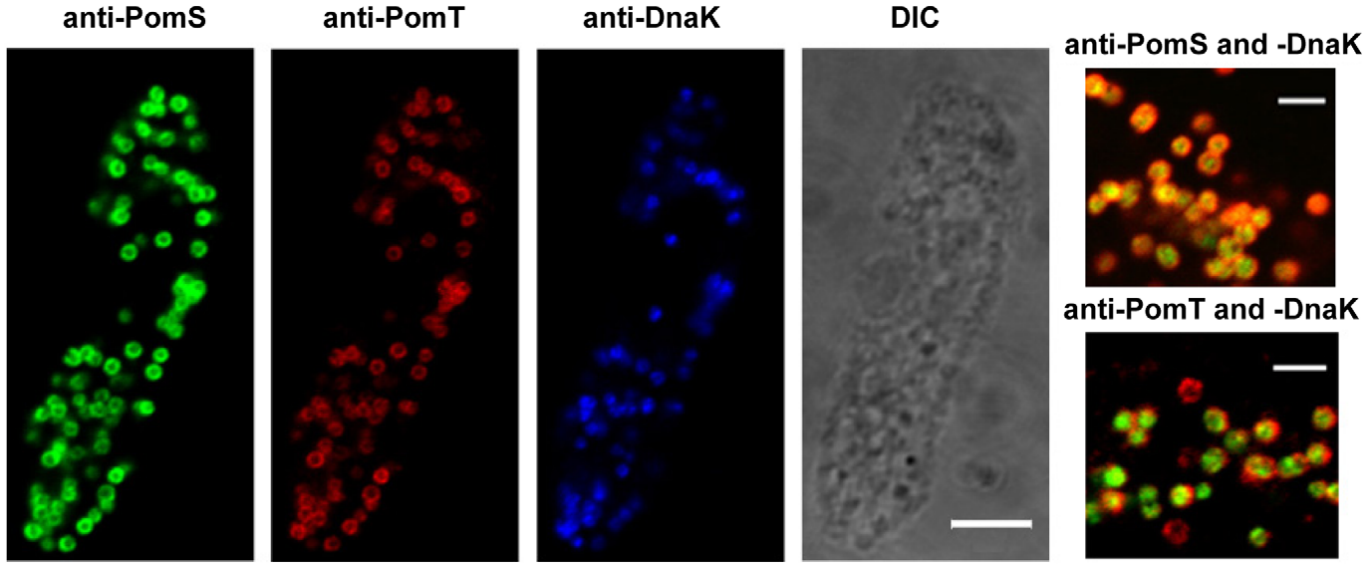
Detection of PomS and PomT in *P. amoebophila* within its natural amoeba host. Left panel: Localization of PomS and PomT by immunofluorescence in an asynchronous culture of *A. castellanii* containing *P. amoebophila*. Halo-shaped fluorescence signals were observed around intracellular *P. amoebophila*. In contrast, signals for the heat-shock protein DnaK were confined to the cytoplasm. No differences were observed for methanol- and PFA- fixed samples. Identical microscopic fields are shown. Bar, 5µm. Right panel: Magnification of intracellular *P. amoebophila*; overlay images of fluorescence signals for PomS and PomT (red), respectively, with DnaK (green) are shown. Bars, 2 µm.

**Figure 4:**
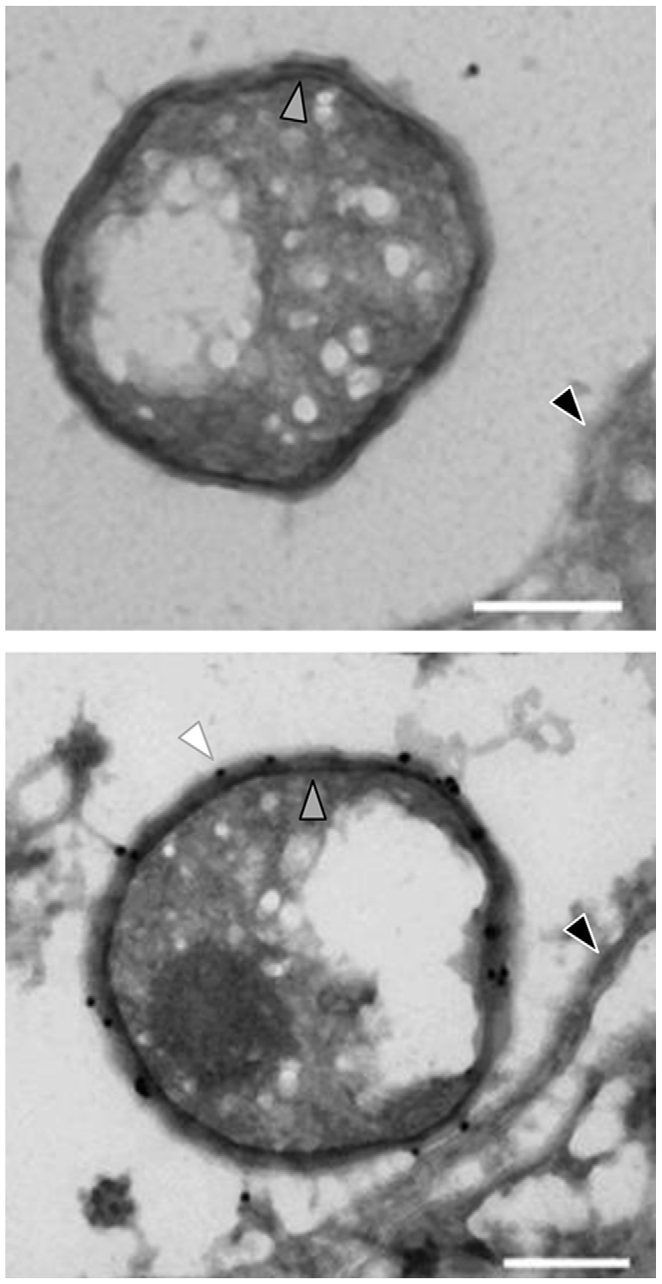
Localization of PomS in the outer membrane of *P. amoebophila* by immuno-transmission electron microscopy. Immunogold labeling with pre-immune serum (left) and polyclonal anti-PomS antibodies (right). Gold particles indicating PomS were confined to the outer membrane of *P. amoebophila*. White arrow head, gold particle in the outer membrane; grey arrowhead, cytoplasmic membrane; black arrowhead, inclusion membrane. Bars, 200 nm.

Immunofluorescence analysis with antibodies targeting PomS and PomT resulted in a halo-shaped fluorescence signal surrounding single *P. amoebophila* cells, demonstrating the presence of these proteins at the periphery of *P. amoebophila* cells (Fig. 3). As *P. amoebophila* is located in single-cell inclusions, it is difficult to distinguish between signals from the outer membrane, the inner membrane and the inclusion membrane by immunofluorescence. However, halo-shaped fluorescence signals were also observed when immunofluorescence analysis was performed with purified EBs. This is a strong evidence for a location of PomS and PomT in the bacterial outer membrane and excludes a location of these proteins in the host-derived inclusion membrane. To further demonstrate the location of the most abundant putative porin PomS in the outer membrane we exploited the higher resolution of immuno-TEM. With this technique, signals for PomS were observed only in the outer membrane of *P. amoebophila*, but not in the cytoplasmic membrane or the inclusion membrane (Fig. 4).

### Transcription and Expression of PomS throughout the Developmental Cycle

**Figure 5:**
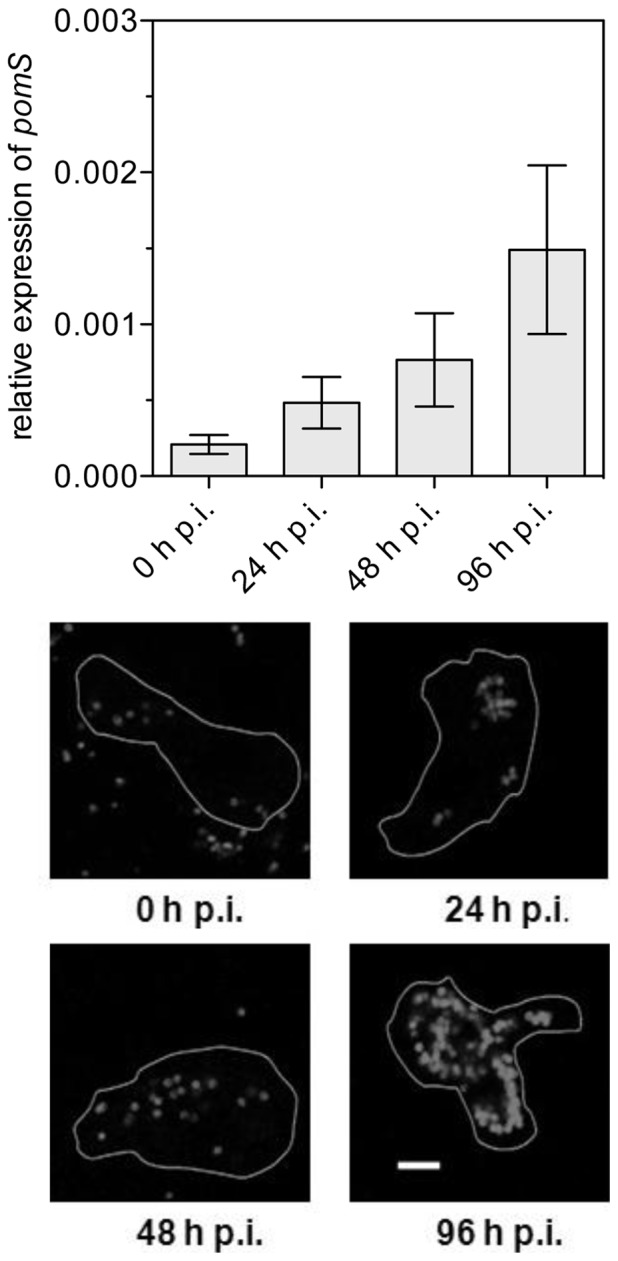
Expression of PomS during the developmental cycle of *P. amoebophila* in its amoeba host. Upper panel: Relative levels of *pomS* transcripts measured by real-time quantitative PCR. *pomS* transcripts were normalized to the 16S rRNA to account for an increase in copy numbers due to multiplication of *P. amoebophila*. Data are shown as the mean of five replicates +/− SEM from a total of three independent infection experiments. Lower panel: Expression of PomS at the same time points as in the upper panel detected by anti-PomS antibody in methanol-fixed cells. Outlines of the amoebae are drawn in white. Bars 5 µm.

To get first insights into the role of PomS during infection and intracellular replication of *P. amoebophila*, we analyzed expression of the gene coding for PomS by real-time quantitative reverse transcription PCR (RT-qPCR) throughout a complete developmental cycle. Expression of *pomS* was detected at all time points during the developmental cycle, with a significant increase from 0 to 48 h p.i. (Mann-Whitney U-test, p≤0.05) and was highest at 96 h p.i. (Fig. 5).

In addition, the presence of PomS in *P. amoebophila* was monitored by immunofluorescence analysis during the developmental cycle. Consistent with our RT-qPCR data, PomS protein was detected at all investigated time points. Fluorescence signal intensities increased, and the halo-shaped signals were better defined at later time points, confirming an elevated expression of PomS at later time points and suggesting an increase of the amount of PomS during the developmental cycle (Fig. 5 and Fig. S1).

### Preincubation with PomS Antibodies does not Alter Infection of Amoebae

**Figure 6:**
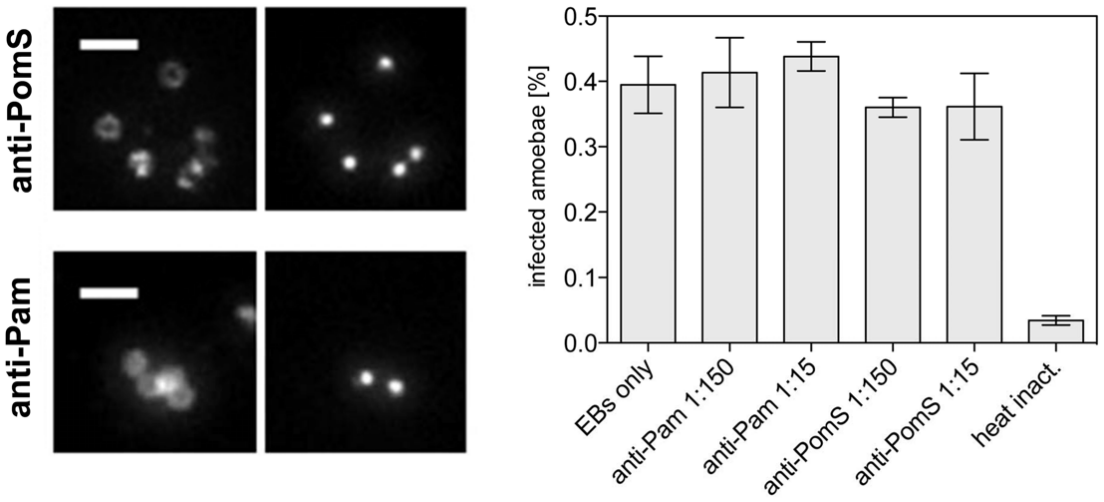
Infection-inhibition assays using anti-Pam and anti-PomS antibodies. Left panel: Incubation of host-free *P. amoebophila* EBs with anti-PomS and anti-Pam antibodies prior to fixation demonstrated that these antibodies can bind unfixed cells. Fluorescence signals derived from specific antibodies (left) and 4′, 6-Diamidino-2-phenylindol (DAPI; right) are shown for identical microscopic fields. Bars, 2 µm. The absence of DAPI signals for some cells indicates cells that lysed during the purification procedure. Right panel: Infection-inhibition assay using preincubations of EBs with anti-Pam and anti-PomS antibodies in different dilutions. The proportion of infected amoebae compared to all counted amoebae of three replicates at 48 h p.i. is shown +/− SEM. Heat-inactivated EBs, used as negative controls, were taken up by the amoebae but did not multiply.

Proteins in the bacterial outer membrane can be important for attachment to and uptake by host cells. As our and previous results identified PomS as a major component of the outer membrane of *P. amoebophila* [36], we tested whether this protein is required for attachment to amoeba host cells and whether infection can be blocked by preincubation of EBs with PomS-specific antibodies. To ensure that the antibodies used in this experiment bind to the outer membrane of unfixed cells, EBs were incubated with the specific antibodies prior to fixation, subsequently fixed and incubated with a secondary antibody. All antibodies were found to bind to the outer membrane of unfixed EBs (Fig. 6). Nevertheless, preincubation with the PomS antibody had no significant effect on bacterial uptake and entry, or on the progress of infection (Fig. 6). Interestingly, also no effect was observed when EBs were preincubated with polyclonal antibodies targeting the immunodominant components of *P. amoebophila* EBs (Fig. 6). High concentrations of this antibody lead to agglutination of EBs at the start of the infection experiment, but even this did not influence the outcome of the infection.

### Porin Function of PomS

**Figure 7:**
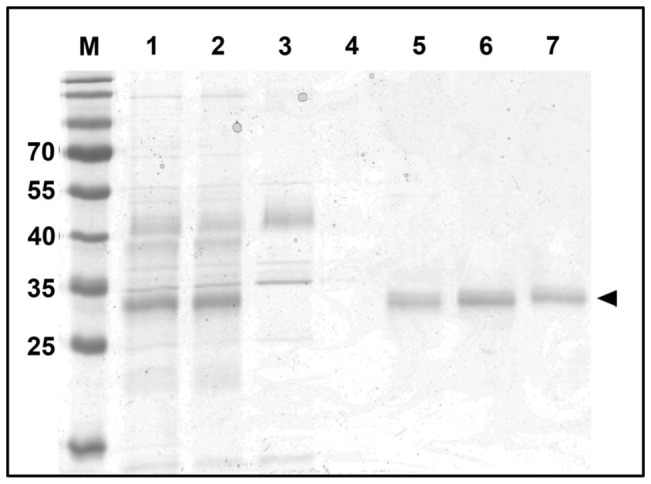
Purification of PomS from *P. amoebophila* EBs. A gel stained with colloidal coomassie is shown; 1, outer membrane fraction after incubation of EBs with n-octyl-POE; 2, outer membrane fraction after precipitation with acetone; 3, column flow through; 4–7, fractions after elution with 0.1, 0.25, 0.3, 0.35 and 0.4 M NaCl. Molecular mass of marker bands (M) in kDa; the expected size of PomS (33.9 kDa) is indicated by an arrow head.

To investigate the putative porin function of PomS we purified this protein directly from *P. amoebophila* EBs. Outer membrane proteins were solubilized with the detergent n-octyl-POE, and DTT was added to reduce disulfide bridges, which are responsible for extensive crosslinking of proteins in the EB cell envelope. A single band at the expected size of PomS in fractions eluted with 250 mM NaCl from an anion exchange column was visible on protein gels, and no other bands were present in this fraction (Fig. 7). This indicates the absence of larger amounts of contaminating protein in this fraction and demonstrates the successful enrichment of PomS. To further analyze this protein fraction, quantitative mass spectrometry analysis was performed. This highly sensitive method identified several *P. amoebophila* proteins. In total, 767 peptide-spectrum matches were assigned to PomS, while only 121 peptide-spectrum matches were assigned to the other *P. amoebophila* proteins. This analysis confirmed that PomS was highly enriched and represented the by far most abundant protein in this fraction (86% based on NSAF quantification). Further proteins included other putative porins and outer membrane proteins with different molecular masses, and at least 18-fold lower abundance (5% and below, based on NSAF) (Table S2).

**Figure 8:**
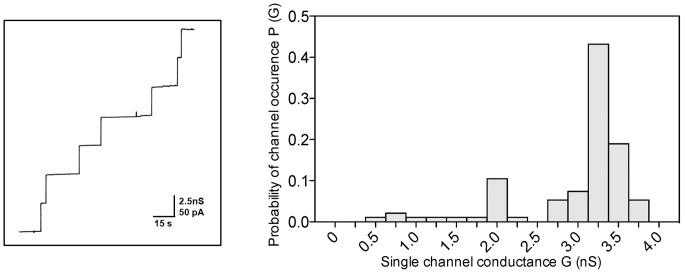
Porin function of purified PomS. Single channel experiments using a PC/*n*-decane membrane in the presence of purified PomS. The aqueous phase contained 1 M KCl and 10 ng ml^−1^ PomS dissolved in 1% Genapol. The applied membrane potential was 20 mV; T = 20°C. Left panel: Single-channel recordings show a uniform stepwise increase as expected for a highly enriched purified porin. Right panel: Frequency of observed conductance increments. P(G) was calculated by dividing the number of fluctuations with a given conductance increment by the total number of conductance fluctuations. Data from both panels suggest that the purified protein fraction contains mainly PomS (about 82% of the total number of pores) and that there is only a very minor contribution of other pores in the histogram (about 18% of the total number of pores) caused either by contaminant porins or by degradation of PomS. The average single-channel conductance was 3.25 nS for 230 single-channel events.

To perform planar lipid bilayer assays purified PomS was added to a lipid membrane consisting of phosphatidylcholine. Single-channel conductance increased in a stepwise fashion indicating that the protein formed defined channels (Fig. 8). The average single-channel conductance was about 3.25 nS in 1 M potassium chloride (KCl). Only a minor fraction of channels (about 18% of the total number of fluctuations) with other conductance was observed suggesting that the protein preparation was essentially free of pore-forming contaminants (Fig. 8). The channels formed by PomS had a long lifetime, similar to porins of other Gram-negative and Gram-positive bacteria [25], [55]–[57]. Analysis of the average single-channel conductance at different KCl concentrations in the aqueous phase showed that the conductance was a nearly linear function of the KCl concentration (Table 2). This is characteristic of many porins of Gram-negative bacteria [25], [26], [47], [57] and suggests that PomS forms a wide and water-filled channel, similar to MOMP of the *Chlamydiaceae*. Lipid bilayer assays were also performed with salts other than KCl to obtain information on the size and selectivity of the channels formed by PomS. Conductance was highest for KCl, followed by lithium chloride (LiCl), and lowest values were observed for potassium acetate (KCH_3_COO, Table 2). Replacement of the Cl^-^ -ion by the less mobile acetate-ion resulted in a stronger decrease in conductance than replacement of the K^+^-ion by the less mobile Li^+^-ion. This means that the influence of anions of different size and mobility on the conductance was more pronounced than that of cations, suggesting anion-selectivity of the PomS channel.

**Table 2:** Average single-channel conductance of PomS in different salt solutions.

Salt	Salt concentration (M)	Single-channel conductance G (nS)
**LiCl**	0.3	1.0
	1.0	2.25
**KCl**	0.01	0.15
	0.03	0.25
	0.10	0.60
	0.30	1.20
	1.0	3.25
	3.0	11
**KCH_3_COO (pH 7)**	0.30	0.60
	1.0	1.50

The membranes were formed from diphytanoyl phosphatidylcholine (PC) dissolved to 1% in n-decane. The aqueous solutions were used unbuffered and had a pH of 6 unless otherwise indicated. The applied voltage was 20 mV, and the temperature was 20°C. The average single-channel conductance, G, was calculated from at least 80 single events.

### Anion-selectivity and Voltage-dependence of PomS

Additional information about the structure of the channel formed by PomS was obtained from zero-current membrane potential measurements in the presence of salt gradients. A 2.5-fold KCl gradient (300 mM versus 750 mM), across a lipid bilayer membrane in which about 100 to 1000 PomS channels were reconstituted, resulted in an asymmetry potential of −7.8 mV at the more dilute side. This result indicated preferential movement of chloride over potassium ions through the pore at neutral pH. The ratio of the chloride permeability, P_Cl_, divided by the potassium permeability, P_K_, was around 2.0, indicating indeed low anion selectivity of the PomS channel. This was further confirmed by measurements with LiCl and potassium acetate; we observed under the same conditions as for KCl, diffusion potentials around −9.5 and −2.6 mV at the more dilute side, respectively (Table 3). The observed selectivity changes dependent on the mobility of the cations and anions indicated that PomS forms a general diffusion pore similar to OmpF and OmpC of *Escherichia coli* [25], [47] and MOMP of *C. psittaci* [28].

**Table 3:** Zero-current membrane potentials of PC/n-decane membranes in the presence of PomS measured for a 2.5-fold gradient of different salts (300 mM versus 750 mM).

Salt	V_m_/mV	P_cation_/P_anion_
KCl	−7.8	0.48
LiCl	−9.5	0.40
KCH_3_COO, pH 7	−2.6	0.79

The zero-current membrane potential V_m_ is defined as the difference between the potential at the dilute side and the potential at the concentrated side. The aqueous salt solutions were used unbuffered and had a pH of 6, if not indicated otherwise; T = 20°C. The permeability ratio P_cation_/P_anion_ was calculated using the Goldman-Hodgkin-Katz equation [46] from at least 3 individual experiments.

**Figure 9:**
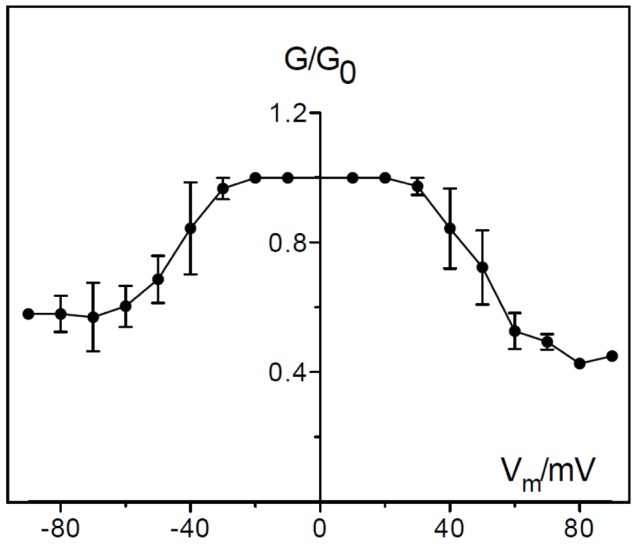
Voltage dependence of PomS. PomS was added in a concentration of 500 ng ml^−1^ to the trans-side side of a PC/*n*-decane membrane in multi-channel experiments. The aqueous phase contained 1 M KCl, pH 6.0. After 30 min the conductance had increased considerably. At this point different potentials were applied to the membrane. The ratio of the conductance G at a given membrane potential (V_m_) divided by the conductance G_o_ at 10 mV was calculated as a function of the membrane potential V_m_ [79]. The membrane potential refers to the cis-side of the membrane. T = 20°C. Means (± SD) of three membranes are shown.

Some porins of Gram-negative bacteria show voltage-dependent closure in reconstitution bilayer experiments [58] although the physiological role of this channel gating is obscure [59]. This effect was also observed for PomS when voltages of positive and negative polarity higher than 30 mV or lower than −30 mV were applied (Fig. 9). The voltage dependence of PomS was essentially unchanged if the protein was added to either the trans- or to the cis-chamber of the membranes. These results indicated a symmetric response of the pore to the applied voltage.

## Discussion

In the *Chlamydiaceae*, the most abundant protein and a major structural component in the outer membrane is the porin MOMP [11], [18]. While two copies of MOMP are encoded in the genome of *Simkania negevensis* [34], no homologue of this protein was found in the two sequenced genomes of members of the *Parachlamydiaceae* [33], [34] – a major difference between this family and its pathogenic relatives from the *Chlamydiaceae*. In a previous study, the presence of a putative novel porin family in *P. amoebophila* has been proposed [36]. Here we show that two members of this porin family, PomS and PomT, are indeed localized in the outer membrane of *P. amoebophila* (Figures 2, 3, 4). PomS, the most abundant outer membrane protein [36], is expressed throughout the developmental cycle with an increase in expression in the later phase of the developmental cycle similar to the expression profile of MOMP in different serovars of *C. trachomatis* (Fig. 5) [60]–[62]. This is consistent with a key function of PomS in the outer membrane of both RBs and EBs.

MOMP is probably not the only factor with respect to attachment to host cells or tissue-specificity in *Chlamydiaceae* [63]. However, preincubations of EBs with antibodies targeting MOMP inhibited infection by either blocking attachment to host cells [64], [65] or at steps after internalization [44], [66]. Preincubation with antibodies targeting the outer membrane proteins OmcB, PorB and members of the Pmp family also reduced infectivity [67]–[70]. In contrast, the infection of amoebae by *P. amoebophila* is not impaired by pre-incubation with anti-PomS or anti-Pam antibodies (Fig. 6). This suggests that neither PomS nor other immunodominant components of the outer membrane of *P. amoebophila* play an important role in attachment to and uptake into amoebae, a process which might be fundamentally different from the uptake into non-phagocytic mammalian cells.

Based on its abundance in the outer membrane it is likely that PomS is an important structural component of the *P. amoebophila* outer membrane. Similar to the *Chlamydiaceae* MOMP it is also a major porin, facilitating transport of small molecules across the outer membrane [24]. The pore formed by PomS is wide, water-filled and anion-selective, presumably because of an excess of positively charged amino acids in or near the pore. The single channel conductance was with 3.25 nS in 1 M KCl relatively high (Fig. 8, Table 3), about 3-times higher than that reported for MOMP which has a single channel conductance of 1 nS in *C. trachomatis* and 1.3 nS in *C. psittaci* [30], [71]. The channels formed by PomS are voltage-dependent in a more or less symmetrical way starting at about ±30 mV (Fig. 9). The voltage dependency of PomS is thus similar to that of the mitochondrial porin VDAC [72], and the channel conducts at high voltages about 50% of its open configuration. Different ion-selectivities have been reported for *Chlamydiaceae* MOMPs. Native MOMP of *C. psittaci* is weakly anion-selective [28], but cation-selective when expressed in *E. coli* [73]. Full length recombinant MOMP of *C. trachomatis* was reported to be either anion- [71] or cation-selective [29], suggesting that tags of the cloning-vector added during the cloning procedure may influence the functional characteristics of these proteins. In this study, ion selectivity was determined using native PomS. Therefore, modifications introduced by recombinant expression can be excluded.

MOMP is highly crosslinked and mostly present as a trimer in *Chlamydiaceae* EBs [30], [54], whereas it is found mostly in its monomeric form in RBs [17]. For PomS we found no evidence for the formation of multimers, which might suggest that this protein is present in the outer membrane of EBs as monomer. Alternative explanations for our observation would be a highly instable PomS multimer that breaks down during purification, or an untypical migration behavior in SDS-PAGE. Trimers of MOMP are stabilized by disulfide-bridges between the monomeric subunits [29], and it has been suggested that opening of the pore is regulated by the reduction of these disulfide bonds [27]. PomS contains only two cysteine residues in contrast to 7–10 cysteine residues in MOMP [74] possibly hindering the formation of stable multimers by PomS and suggesting a different opening mechanism for this pore.

Whether the other members of the PomS protein family also function as porins has to be determined. The predicted beta-barrel structure of PomT and its location in the outer membrane strongly suggests a porin function. PomU is not predicted to form a beta-barrel, and PomV represents a lipoprotein according to *in silico* analysis [48]. However, all proteins of this family were predicted to encode a signal peptide and showed a pronounced toxic effect on *E. coli* when expression of the full length constructs was induced (Fig. 1). This is consistent with the notion that outer membrane proteins, and porins in particular, are generally difficult to overexpress in *E. coli*, because if properly folded, they might insert into the outer membrane, and their pore-forming activity can be toxic for the host [49], [75].

The different members of the PomS protein family could play a role in the adaption to different environmental conditions as they show an only low degree of amino acid sequence identity (22–28%) and hence likely differ in pore size, uptake rates or ion specificity. Well-studied examples of homologous yet differentially sized porins are OmpF and OmpC of *E. coli* whose levels of expression depends on the osmolarity of the extracellular milieu [76], [77]. The smaller pore formed by OmpC is dominant under conditions of high osmolarity whereas OmpF is upregulated under iso- or hypoosmotic conditions (Benz et al., 1985). Expansions of genes encoding porins are apparent in several chlamydial genomes. In the *Chlamydiaceae*, the protein PorB was identified as putative porin based on its weak sequence similarity to MOMP by genome analysis; its pore-forming function was later confirmed [70]. The much larger genomes of *W. chondrophila* and *S. negevensis* encode 11 and 35 MOMP-like genes, respectively, which are more diverged from known *Chlamydiaceae* MOMPs [34], [78]. Within the chlamydiae, the PomS protein family analyzed here seems to be restricted to *P. amoebophila* and *Parachlamydia acanthamoebae* and lacks close homologues in other bacteria. The *P. amoebophila* PomS corresponds to the MOMP of the *Chlamydiaceae* with respect to abundance in the outer membrane and its function as major porin, and it thus represents a specific adaptation of this amoeba symbiont.

## Supporting Information

Figure S1 **Fluorescence intensity derived from anti-PomS antibodies increases during infection.** Quantification of fluorescence intensity was performed using the image analysis software daime [86]. The mean fluorescence intensity (± SD) is shown for each time point.
(TIF)

Table S1 **Primers used for qPCR targeting genes of** ***P. amoebophila.***
(DOCX)

Table S2 **Quantification by mass spectrometry shows that PomS is highly enriched in purified porin fractions.** The percent abundance of proteins with five or more assigned spectra was calculated based on the normalized spectral abundance factor (NSAF) [38], [39].
(DOCX)

## References

[1] Longbottom D, Coulter LJ (2003) Animal chlamydioses and zoonotic implications. J Comp Pathol 128: 217–244. 12834606 10.1053/jcpa.2002.0629

[2] Horn M (2008) Chlamydiae as symbionts in eukaryotes. Ann Rev Microbiol 62: in press. 10.1146/annurev.micro.62.081307.162818 18473699

[3] Blasi F, Tarsia P, Aliberti S (2009) *Chlamydophila pneumoniae* . Clinical Microbiology and Infection 15: 29–35. 10.1111/j.1469-0691.2008.02130.x 19220337

[4] Bébéar C, De Barbeyrac B (2009) Genital *Chlamydia trachomatis* infections. Clinical Microbiology and Infection 15: 4–10. 10.1111/j.1469-0691.2008.02647.x 19220334

[5] Abdelrahman YM, Belland RJ (2005) The chlamydial developmental cycle. FEMS Microbiol Rev 29: 949–959. 16043254 10.1016/j.femsre.2005.03.002

[6] Hackstadt T, Fischer ER, Scidmore MA, Rockey DD, Heinzen RA (1997) Origins and functions of the chlamydial inclusion. Trends in Microbiology 5: 288–293. 9234512 10.1016/S0966-842X(97)01061-5

[7] Hybiske K, Stephens RS (2007) Mechanisms of host cell exit by the intracellular bacterium *Chlamydia* . Proc Natl Acad Sci U S A 104: 11430–11435. 17592133 10.1073/pnas.0703218104 PMC2040915

[8] Pal S, Theodor I, Peterson EM, de la Maza LM (1997) Immunization with an acellular vaccine consisting of the outer membrane complex of *Chlamydia trachomatis* induces protection against a genital challenge. Infect Immun 65: 3361–3369. 9234798 10.1128/iai.65.8.3361-3369.1997 PMC175475

[9] Tan TW, Herring AJ, Anderson IE, Jones GE (1990) Protection of sheep against *Chlamydia psittaci* infection with a subcellular vaccine containing the major outer membrane protein. Infect Immun 58: 3101–3108. 2387636 10.1128/iai.58.9.3101-3108.1990 PMC313617

[10] Everett KD, Hatch TP (1995) Architecture of the cell envelope of *Chlamydia psittaci* 6BC. J Bacteriol 177: 877–882. 7532170 10.1128/jb.177.4.877-882.1995 PMC176678

[11] Hatch TP, Vance DW Jr, Al-Hossainy E (1981) Identification of a major envelope protein in *Chlamydia* spp. J Bacteriol 146: 426–429. 7217005 10.1128/jb.146.1.426-429.1981 PMC217103

[12] Raulston JE (1995) Chlamydial envelope components and pathogen-host cell interactions. Molecular Microbiology 15: 607–616. 7783633 10.1111/j.1365-2958.1995.tb02370.x

[13] Hatch T (1996) Disulfide cross-linked envelope proteins: the functional equivalent of peptidoglycan in chlamydiae? J Bacteriol 178: 1–5. 8550401 10.1128/jb.178.1.1-5.1996 PMC177613

[14] Salari SH, Ward ME (1981) Polypeptide composition of *Chlamydia trachomatis* . J Gen Microbiol 123: 197–207. 7320696 10.1099/00221287-123-2-197

[15] Tanzer RJ, Hatch TP (2001) Characterization of outer membrane proteins in *Chlamydia trachomatis* LGV serovar L2. J Bacteriol 183: 2686–2690. 11274132 10.1128/JB.183.8.2686-2690.2001 PMC95189

[16] Liu X, Afrane M, Clemmer DE, Zhong G, Nelson DE (2010) Identification of *Chlamydia trachomatis* Outer Membrane Complex Proteins by Differential Proteomics. Journal of Bacteriology 192: 2852–2860. 20348250 10.1128/JB.01628-09 PMC2876478

[17] Hatch TP, Allan I, Pearce JH (1984) Structural and polypeptide differences between envelopes of infective and reproductive life cycle forms of *Chlamydia* spp. J Bacteriol 157: 13–20. 6690419 10.1128/jb.157.1.13-20.1984 PMC215122

[18] Caldwell HD, Kromhout J, Schachter J (1981) Purification and partial characterization of the major outer membrane protein of *Chlamydia trachomatis* . Infect Immun 31: 1161–1176. 7228399 10.1128/iai.31.3.1161-1176.1981 PMC351439

[19] McCoy AJ, Maurelli AT (2006) Building the invisible wall: updating the chlamydial peptidoglycan anomaly. Trends Microbiol 14: 70–77. 16413190 10.1016/j.tim.2005.12.004

[20] Hatch TP, Miceli M, Sublett JE (1986) Synthesis of disulfide-bonded outer membrane proteins during the developmental cycle of *Chlamydia psittaci* and *Chlamydia trachomatis* . J Bacteriol 165: 379–385. 3944054 10.1128/jb.165.2.379-385.1986 PMC214428

[21] Hackstadt T, Todd WJ, Caldwell HD (1985) Disulfide-mediated interactions of the chlamydial major outer membrane protein: role in the differentiation of chlamydiae? J Bacteriol 161: 25–31. 2857160 10.1128/jb.161.1.25-31.1985 PMC214830

[22] Newhall WJ (1987) Biosynthesis and disulfide cross-linking of outer membrane components during the growth cycle of *Chlamydia trachomatis* . Infect Immun 55: 162–168. 3793227 10.1128/iai.55.1.162-168.1987 PMC260295

[23] Stephens RS, Tam MR, Kuo CC, Nowinski RC (1982) Monoclonal antibodies to *Chlamydia trachomatis*: antibody specificities and antigen characterization. J Immunol 128: 1083–1089. 7035557

[24] Nikaido H (2003) Molecular basis of bacterial outer membrane permeability revisited. Microbiol Mol Biol Rev 67: 593–656. 14665678 10.1128/MMBR.67.4.593-656.2003 PMC309051

[25] Benz R (1994) Solute uptake through bacterial outer membrane. In Bacterial cell wall.; Ghuysen JM, Hakenbeck, R, editor. Amsterdam: Elsevier Science B. V.

[26] Benz R, Bauer K (1988) Permeation of hydrophilic molecules through the outer membrane of gram-negative bacteria. Eur J Biochem 176 1–19. 10.1111/j.1432-1033.1988.tb14245.x 2901351

[27] Bavoil P, Ohlin A, Schachter J (1984) Role of disulfide bonding in outer membrane structure and permeability in *Chlamydia trachomatis* . Infect Immun 44: 479–485. 6715046 10.1128/iai.44.2.479-485.1984 PMC263545

[28] Wyllie S, Ashley RH, Longbottom D, Herring AJ (1998) The major outer membrane protein of *Chlamydia psittaci* functions as a porin-like ion channel. Infect Immun 66: 5202–5207. 9784523 10.1128/iai.66.11.5202-5207.1998 PMC108649

[29] Findlay H, McClafferty H, Ashley R (2005) Surface expression, single-channel analysis and membrane topology of recombinant *Chlamydia trachomatis* Major Outer Membrane Protein. BMC Microbiology 5: 5. 15673471 10.1186/1471-2180-5-5 PMC549562

[30] Sun G, Pal S, Sarcon AK, Kim S, Sugawara E, et al (2007) Structural and Functional Analyses of the Major Outer Membrane Protein of *Chlamydia trachomatis* . Journal of Bacteriology 189: 6222–6235. 17601785 10.1128/JB.00552-07 PMC1951919

[31] Newhall V, Wilbert JJ, Robert B (1983) Disulfide-linked oligomers of the Major Outer Membrane Protein of *Chlamydiae* . J Bacteriol 154: 998–1001. 6841322 10.1128/jb.154.2.998-1001.1983 PMC217558

[32] Heinz E, Rockey DD, Montanaro J, Aistleitner K, Wagner M, et al (2010) Inclusion Membrane Proteins of *Protochlamydia amoebophila* UWE25 Reveal a Conserved Mechanism for Host Cell Interaction among the *Chlamydiae* . J Bacteriol 192: 5093–5102. 20675479 10.1128/JB.00605-10 PMC2944539

[33] Horn M, Collingro A, Schmitz-Esser S, Beier CL, Purkhold U, et al (2004) Illuminating the evolutionary history of chlamydiae. Science 304: 728–730. 15073324 10.1126/science.1096330

[34] Collingro A, Tischler P, Weinmaier T, Penz T, Heinz E, et al (2011) Unity in Variety–The Pan-Genome of the *Chlamydiae* . Molecular Biology and Evolution 28: 3253–3270. 21690563 10.1093/molbev/msr161 PMC3247790

[35] Collingro A, Toenshoff ER, Taylor MW, Fritsche TR, Wagner M, et al (2005) ‘*Candidatus* Protochlamydia amoebophila’, an endosymbiont of *Acanthamoeba* spp. Int J Syst Evol Microbiol 55: 1863–1866. 16166679 10.1099/ijs.0.63572-0

[36] Heinz E, Pichler P, Heinz C, op den Camp HJM, Toenshoff ER, et al (2010) Proteomic analysis of the outer membrane of *Protochlamydia amoebophila* elementary bodies. PROTEOMICS 10: 4363–4376. 21136591 10.1002/pmic.201000302

[37] Heinz C, Roth E, Niederweis M (2003) Purification of Porins from *Mycobacterium smegmatis*. 139–150. 10.1385/1-59259-400-X:139 12824550

[38] Zybailov B, Mosley AL, Sardiu ME, Coleman MK, Florens L, et al (2006) Statistical Analysis of Membrane Proteome Expression Changes in *Saccharomyces cerevisiae* . Journal of Proteome Research 5: 2339–2347. 16944946 10.1021/pr060161n

[39] Collier TS, Sarkar P, Franck WL, Rao BM, Dean RA, et al (2010) Direct Comparison of Stable Isotope Labeling by Amino Acids in Cell Culture and Spectral Counting for Quantitative Proteomics. Analytical Chemistry 82: 8696–8702. 20845935 10.1021/ac101978b

[40] Untergasser A, Nijveen H, Rao X, Bisseling T, Geurts R, et al (2007) Primer3Plus, an enhanced web interface to Primer3. Nucl Acids Res 35: W71–74. 17485472 10.1093/nar/gkm306 PMC1933133

[41] Borges V, Ferreira R, Nunes A, Nogueira P, Borrego MJ, et al (2010) Normalization strategies for real-time expression data in *Chlamydia trachomatis* . Journal of Microbiological Methods 82: 256–264. 20619305 10.1016/j.mimet.2010.06.013

[42] Ritter K (1991) Affinity purification of antibodies from sera using polyvinylidenedifluoride (PVDF) membranes as coupling matrices for antigens presented by autoantibodies to triosephosphate isomerase. Journal of Immunological Methods 137: 209–215. 1849541 10.1016/0022-1759(91)90026-c

[43] Abeyrathne PD, Lam JS (2007) Conditions that allow for effective transfer of membrane proteins onto nitrocellulose membrane in Western blots. Can J Microbiol 53: 526–532. 17612609 10.1139/W07-007

[44] Caldwell HD, Perry LJ (1982) Neutralization of *Chlamydia trachomatis* infectivity with antibodies to the major outer membrane protein. Infect Immun 38: 745–754. 7141712 10.1128/iai.38.2.745-754.1982 PMC347801

[45] Benz R, Janko K, Boos W, Läuger P (1978) Formation of large, ion-permeable membrane channels by the matrix protein (porin) of *Escherichia coli* . Biochim Biophys Acta 511: 305–319. 356882 10.1016/0005-2736(78)90269-9

[46] Benz R, Janko K, Läuger P (1979) Ionic selectivity of pores formed by the matrix protein (porin) of *Escherichia coli* . Biochim Biophys Acta 551: 238–247. 369608 10.1016/0005-2736(89)90002-3

[47] Benz R, Schmid A, Hancock RE (1985) Ion selectivity of gram-negative bacterial porins. Journal of Bacteriology 162: 722–727. 2580824 10.1128/jb.162.2.722-727.1985 PMC218910

[48] Heinz E, Tischler P, Rattei T, Myers G, Wagner M, et al (2009) Comprehensive in silico prediction and analysis of chlamydial outer membrane proteins reflects evolution and life style of the *Chlamydiae* . BMC Genomics 10: 634. 20040079 10.1186/1471-2164-10-634 PMC2811131

[49] Koehler JE, Birkelund S, Stephens RS (1992) Overexpression and surface localization of the *Chlamydia trachomatis* major outer membrane protein in *Escherichia coli* . Mol Microbiol 6: 1087–1094. 1588812 10.1111/j.1365-2958.1992.tb01545.x

[50] Miroux B, Walker JE (1996) Over-production of Proteins in *Escherichia coli*: Mutant Hosts that Allow Synthesis of some Membrane Proteins and Globular Proteins at High Levels. Journal of Molecular Biology 260: 289–298. 8757792 10.1006/jmbi.1996.0399

[51] Hoffmann F, Rinas U (2004) Stress Induced by Recombinant Protein Production in *Escherichia coli*. Physiological Stress Responses in Bioprocesses: Springer Berlin/Heidelberg. 73–92. 10.1007/b93994 15217156

[52] Iost I, Guillerez J, Dreyfus M (1992) Bacteriophage T7 RNA polymerase travels far ahead of ribosomes in vivo. J Bacteriol 174: 619–622. 1729251 10.1128/jb.174.2.619-622.1992 PMC205757

[53] Welte W, Nestel U, Wacker T, Diederichs K (1995) Structure and function of the porin channel. Kidney Int 48: 930–940. 8569102 10.1038/ki.1995.374

[54] McCafferty MC, Herring AJ, Andersen AA, Jones GE (1995) Electrophoretic analysis of the major outer membrane protein of *Chlamydia psittaci* reveals multimers which are recognized by protective monoclonal antibodies. Infect Immun 63: 2387–2389. 7768628 10.1128/iai.63.6.2387-2389.1995 PMC173318

[55] Trias J, Benz R (1994) Permeability of the cell wall of *Mycobacterium smegmatis* . Molecular Microbiology 14: 283–290. 7830572 10.1111/j.1365-2958.1994.tb01289.x

[56] Trias J, Jarlier V, Benz R (1992) Porins in the cell wall of mycobacteria. Science 258: 1479–1481. 1279810 10.1126/science.1279810

[57] Benz R (2001) Porins - structure and function; Winkelmann G, editor. Weinheim: Wiley-VCH.

[58] Schindler H, Rosenbusch JP (1978) Matrix protein from *Escherichia coli* outer membranes forms voltage-controlled channels in lipid bilayers. Proceedings of the National Academy of Sciences 75: 3751–3755. 10.1073/pnas.75.8.3751 PMC392864 358202

[59] Sen K, Hellman J, Nikaido H (1988) Porin channels in intact cells of *Escherichia coli* are not affected by Donnan potentials across the outer membrane. Journal of Biological Chemistry 263: 1182–1187. 2447086

[60] Gomes JP, Hsia RC, Mead S, Borrego MJ, Dean D (2005) Immunoreactivity and differential developmental expression of known and putative *Chlamydia trachomatis* membrane proteins for biologically variant serovars representing distinct disease groups. Microbes Infect 7: 410–420. 15784185 10.1016/j.micinf.2004.11.014

[61] Belland RJ, Zhong G, Crane DD, Hogan D, Sturdevant D, et al (2003) Genomic transcriptional profiling of the developmental cycle of *Chlamydia trachomatis* . Proc Natl Acad Sci U S A 100: 8478–8483. 12815105 10.1073/pnas.1331135100 PMC166254

[62] Albrecht M, Sharma CM, Reinhardt R, Vogel J, Rudel T (2010) Deep sequencing-based discovery of the *Chlamydia trachomatis* transcriptome. Nucleic Acids Research 38: 868–877. 19923228 10.1093/nar/gkp1032 PMC2817459

[63] Stothard DR, Boguslawski G, Jones RB (1998) Phylogenetic Analysis of the *Chlamydia trachomatis* Major Outer Membrane Protein and Examination of Potential Pathogenic Determinants. Infect Immun 66: 3618–3625. 9673241 10.1128/iai.66.8.3618-3625.1998 PMC108394

[64] Su H, Caldwell HD (1991) In vitro neutralization of *Chlamydia trachomatis* by monovalent Fab antibody specific to the major outer membrane protein. Infect Immun 59: 2843–2845. 1713202 10.1128/iai.59.8.2843-2845.1991 PMC258096

[65] Ward ME, Murray A (1984) Control mechanisms governing the infectivity of *Chlamydia trachomatis* for HeLa cells: mechanisms of endocytosis. J Gen Microbiol 130: 1765–1780. 6470672 10.1099/00221287-130-7-1765

[66] Peeling R, Maclean IW, Brunham RC (1984) In vitro neutralization of *Chlamydia trachomatis* with monoclonal antibody to an epitope on the major outer membrane protein. Infect Immun 46: 484–488. 6209221 10.1128/iai.46.2.484-488.1984 PMC261559

[67] Moelleken K, Hegemann JH (2008) The *Chlamydia* outer membrane protein OmcB is required for adhesion and exhibits biovar-specific differences in glycosaminoglycan binding. Mol Microbiol 67: 403–419. 18086188 10.1111/j.1365-2958.2007.06050.x PMC2229832

[68] Wehrl W, Brinkmann V, Jungblut PR, Meyer TF, Szczepek AJ (2004) From the inside out–processing of the Chlamydial autotransporter PmpD and its role in bacterial adhesion and activation of human host cells. Mol Microbiol 51: 319–334. 14756775 10.1046/j.1365-2958.2003.03838.x

[69] Mölleken K, Schmidt E, Hegemann JH (2010) Members of the Pmp protein family of *Chlamydia pneumoniae* mediate adhesion to human cells via short repetitive peptide motifs. Molecular Microbiology 78: 1004–1017. 21062373 10.1111/j.1365-2958.2010.07386.x PMC2997323

[70] Kubo A, Stephens RS (2000) Characterization and functional analysis of PorB, a *Chlamydia* porin and neutralizing target. Mol Microbiol 38: 772–780. 11115112 10.1046/j.1365-2958.2000.02167.x

[71] Hughes ES, Shaw KM, Ashley RH (2001) Mutagenesis and functional reconstitution of chlamydial Major Outer Membrane Proteins: VS4 domains are not tequired for pore formation but modify channel function. Infect Immun 69: 1671–1678. 11179342 10.1128/IAI.69.3.1671-1678.2001 PMC98071

[72] Benz R (1994) Permeation of hydrophilic solutes through mitochondrial outer membranes: review on mitochondrial porins. Biochimica et Biophysica Acta (BBA) - Reviews on Biomembranes 1197: 167–196. 8031826 10.1016/0304-4157(94)90004-3

[73] Wyllie S, Longbottom D, Herring AJ, Ashley RH (1999) Single channel analysis of recombinant major outer membrane protein porins from *Chlamydia psittaci* and *Chlamydia pneumoniae* . FEBS Lett 445: 192–196. 10069399 10.1016/s0014-5793(99)00121-0

[74] Stephens RS (1999) Chlamydia. Washington DC: ASM Press.

[75] Wagner S, Bader ML, Drew D, de Gier J-W (2006) Rationalizing membrane protein overexpression. Trends in Biotechnology 24: 364–371. 16820235 10.1016/j.tibtech.2006.06.008

[76] Alphen WV, Lugtenberg B (1977) Influence of osmolarity of the growth medium on the outer membrane protein pattern of *Escherichia coli* . J Bacteriol 131: 623–630. 328490 10.1128/jb.131.2.623-630.1977 PMC235471

[77] Pratt LA, Hsing W, Gibson KE, Silhavy TJ (1996) From acids to osmZ: multiple factors influence synthesis of the OmpF and OmpC porins in *Escherichia coli* . Molecular Microbiology 20: 911–917. 8809744 10.1111/j.1365-2958.1996.tb02532.x

[78] Bertelli C, Collyn F, Croxatto A, Rückert C, Polkinghorne A, et al (2010) The *Waddlia* Genome: A Window into Chlamydial Biology. PLoS ONE 5: e10890. 20531937 10.1371/journal.pone.0010890 PMC2878342

[79] Ludwig O, De Pinto V, Palmieri F, Benz R (1986) Pore formation by the mitochondrial porin of rat brain in lipid bilayer membranes. Biochim Biophys Acta 860: 268–276. 2427116 10.1016/0005-2736(86)90523-7

[80] Petersen TN, Brunak S, von Heijne G, Nielsen H (2011) SignalP 4.0: discriminating signal peptides from transmembrane regions. Nat Meth 8: 785–786. 10.1038/nmeth.1701 21959131

[81] Rey S, Acab M, Gardy JL, Laird MR, deFays K, et al (2005) PSORTdb: a protein subcellular localization database for bacteria. Nucleic Acids Res 33: D164–168. 15608169 10.1093/nar/gki027 PMC539981

[82] Bagos PG, Liakopoulos TD, Hamodrakas SJ (2004) Finding beta-barrel outer membrane proteins with a markov chain model. WSEAS Transactions on Biology and Biomedicine 2: 186–189.

[83] Bagos PG, Liakopoulos TD, Spyropoulos IC, Hamodrakas SJ (2004) PRED-TMBB: a web server for predicting the topology of beta-barrel outer membrane proteins. Nucleic Acids Res 32: W400–404. 15215419 10.1093/nar/gkh417 PMC441555

[84] Juncker AS, Willenbrock H, Von Heijne G, Brunak S, Nielsen H, et al (2003) Prediction of lipoprotein signal peptides in Gram-negative bacteria. Protein Sci 12: 1652–1662. 12876315 10.1110/ps.0303703 PMC2323952

[85] Remmert M, Linke D, Lupas AN, Söding J (2009) HHomp–prediction and classification of outer membrane proteins. Nucleic Acids Research 37: W446–W451. 19429691 10.1093/nar/gkp325 PMC2703889

[86] Daims H, Lücker S, Wagner M (2006) daime, a novel image analysis program for microbial ecology and biofilm research. Environmental Microbiology 8: 200–213. 16423009 10.1111/j.1462-2920.2005.00880.x

