# Supplementary figures and images for: Identification and Characterization of a Novel Porin Family Highlights a Major Difference in the Outer Membrane of Chlamydial Symbionts and Pathogens
Source: PLoS One. 2013 Jan 31;8(1):e55010. doi: 10.1371/journal.pone.0055010 (PMC3561449; doi:10.1371/journal.pone.0055010)

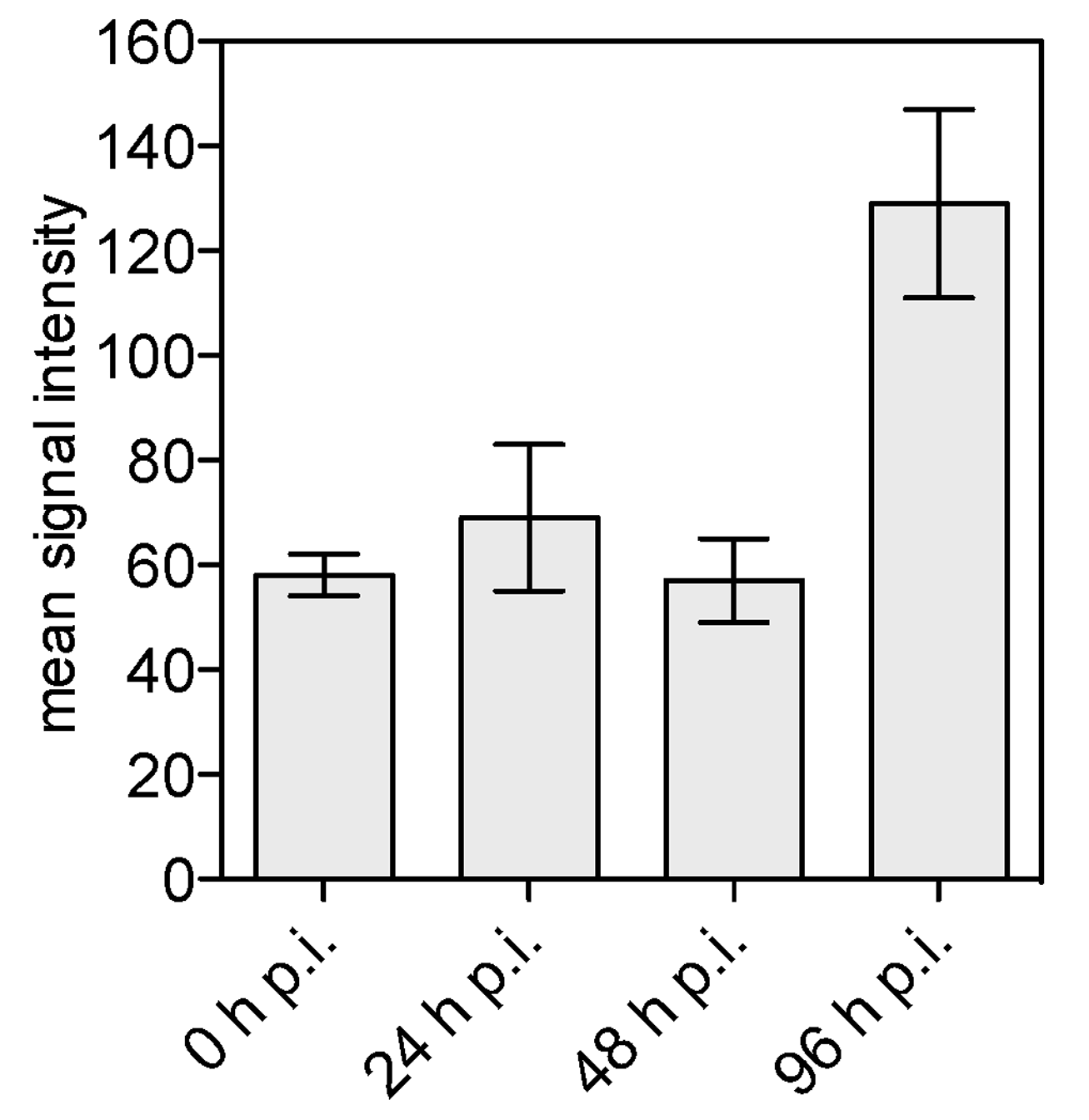

Supplement: Figure S1 — Fluorescence intensity derived from anti-PomS antibodies increases during infection. Quantification of fluorescence intensity was performed using the image analysis software daime [86]. The mean fluorescence intensity (± SD) is shown for each time point. (TIF) [file pone.0055010.s001.tif]
