# Supplementary material for: Identification and Characterization of a Novel Porin Family Highlights a Major Difference in the Outer Membrane of Chlamydial Symbionts and Pathogens
Source: PLoS One. 2013 Jan 31;8(1):e55010. doi: 10.1371/journal.pone.0055010 (PMC3561449; doi:10.1371/journal.pone.0055010)
Supplement: Table S1 — Primers used for qPCR targeting genes of P. amoebophila. (DOCX) [file pone.0055010.s002.docx]

**Table S1: Primers used for qPCR targeting genes of *P. amoebophila***

| Primer | Target molecule | Primer sequence  (5’-3’) | Primer location | | Size of amplicon | |
| --- | --- | --- | --- | --- | --- | --- |
| q16S_F | 16S rRNA gene | GCA AGT CGA ACG AAA CCT C | 30-47 | | 88 bp | |
| q16S_R | 16S rRNA gene | TTC CAA CCG TTA TCC CAG AG | 98-118 |  | |  |
|  |  |  |  | |  | |
| q1489_F | pc1489 gene | TGG AAC CAA AAA GCT CCA TC | 118-138 | | 143 bp | |
| q1489_R | pc1489 gene | CAG CCC CAA TTT ACA GAA GC | 241-261 | |  | |
|  |  |  |  | |  | |
