# Supplementary material for: Identification and Characterization of a Novel Porin Family Highlights a Major Difference in the Outer Membrane of Chlamydial Symbionts and Pathogens
Source: PLoS One. 2013 Jan 31;8(1):e55010. doi: 10.1371/journal.pone.0055010 (PMC3561449; doi:10.1371/journal.pone.0055010)
Supplement: Table S2 — Quantification by mass spectrometry shows that PomS is highly enriched in purified porin fractions. The percent abundance of proteins with five or more assigned spectra was calculated based on the normalized spectral abundance factor (NSAF) [38], [39]. (DOCX) [file pone.0055010.s003.docx]

**Table S2: Quantification by mass spectrometry shows that PomS is highly enriched in purified porin fractions.** The percent abundance of proteins with five or more assigned spectra was calculated based on the normalized spectral abundance factor (NSAF) [[38](#_ENREF_38),[39](#_ENREF_39)].

| Locus tag | Protein description | Protein mass (kDa) | Protein length (aa) | Spectral counts | NSAF | % abundance | Fold lower than PomS |
| --- | --- | --- | --- | --- | --- | --- | --- |
| pc1489 | PomS | 36.3 | 317 | 767 | 2.42 | 85.7 | 1.0 |
| pc1885 | hyp. protein | 24.5 | 217 | 29 | 0.13 | 4.7 | 18.1 |
| pc1077 | PomT | 39.0 | 345 | 44 | 0.13 | 4.5 | 19.0 |
| pc0675 | hyp. protein | 37.3 | 320 | 28 | 0.09 | 3.1 | 27.7 |
| pc0575 | hyp. protein | 40.3 | 342 | 8 | 0.02 | 0.8 | 103.4 |
| pc1860 | PomV | 37.5 | 325 | 7 | 0.02 | 0.8 | 112.3 |
| pc0004 | hyp. protein | 53.8 | 470 | 5 | 0.01 | 0.4 | 227.4 |
